# Early-Onset Neonatal Sepsis in Turkey: A Single-Center 7-Year Experience in Etiology and Antibiotic Susceptibility

**DOI:** 10.3390/children9111642

**Published:** 2022-10-28

**Authors:** Sevilay Topcuoglu, Salih Demirhan, Emre Dincer, Elif Ozalkaya, Guner Karatekin

**Affiliations:** Zeynep Kamil Maternity and Children’s Hospital, University of Health Sciences, Istanbul 34668, Turkey

**Keywords:** early-onset neonatal sepsis, mortality, antibiotic susceptibility

## Abstract

Background: The pathogen distribution and antibiotic susceptibility of the pathogens in early-onset sepsis (EOS) differ between countries. The epidemiological data from a limited number of studies about EOS in Turkey are insufficient. In this study, we aimed to evaluate the culture-proven EOS cases, causative microorganisms, antibiotic susceptibility patterns, and risk factors for mortality in EOS. Methods: This is a retrospective, single-center study over a 7-year period, from 2013 to 2020, at Zeynep Kamil Maternity and Children’s Hospital, İstanbul, Turkey. Results: During the study period, 8229 newborns were admitted to our neonatal intensive care unit. Culture-proven EOS was detected in 101 patients (0.12%). Out of these, 56 (55.4%) were Gram-positive, and 45 (44.5%) were Gram-negative sepsis. The most common isolated organism was *E. coli* (28.7%, *n* = 29), followed by GBS (16.8%, *n* = 17) and *S. aureus* (15.8%, *n* = 16). An ampicillin and gentamicin combination had antimicrobial coverage in 92.6% of cases. Seventeen patients (16.8%) died because of EOS. Severe neutropenia was found to be an independent risk factor for mortality in EOS (*p* = 0.001, OR = 14.4, CI 95%: 2.8–74). Conclusions: Although the majority of causative agents were Gram-positive (55.4%), the most common isolated organism was *E. coli.* An empirical antibiotic regimen of ampicillin and gentamicin continues to have an adequate coverage for EOS in our population.

## 1. Introduction

Early-onset sepsis (EOS) is still one of the most common causes of morbidity and mortality in the neonatal period [1]. The mortality rate substantially increases with lower birth weight and gestational age [1,2]. After implementing intrapartum antibiotic prophylaxis, the incidence and mortality of EOS have decreased significantly in the past decades, especially in developed countries [1,2]. The incidence of EOS was 0.32–0.97 per 1000 live births in Europe, comparable to reports from the USA (1.08/1000) and Australia (0.67/1000) [1,2,3,4,5]. However, its incidence remains higher in developing countries, where data is scarce [6,7]. In a systematic review by Khalil et al., EOS incidence in middle-income countries was 3.3–15.7 per 1000 live births in the Middle East region [6].

The etiology of EOS has changed dramatically after implementing guidelines for routine group B *Streptococcus* (GBS) screening. Although it has been shown that *E. coli* has become the most frequently detected pathogen in EOS of extremely preterm infants, the leading microorganism for EOS is still GBS in developed countries [4,8]. Evidence indicates that the pathogen distribution of EOS differs among developing and middle-income countries in the Middle East region and Asia, where GBS is less frequently reported, while Gram-negative pathogens, such as *Klebsiella pneumoniae* and *E. coli*, are the major pathogens and are less likely to be susceptible to currently recommended empirical antibiotic therapy [6,7,9].

The epidemiological data from a limited number of studies about EOS in Turkey are insufficient. In this study, we aimed to evaluate the culture-proven EOS cases, causative microorganisms, antibiotic susceptibility patterns, and risk factors for mortality in EOS.

## 2. Materials and Methods

### 2.1. Settings and Study Population

This is a single-center, retrospective study over 7-year period from August 2013 to August 2020. Our unit is a 63-bed neonatal intensive care unit (NICU) of a tertiary hospital, with approximately 1200 annual admissions. We evaluated all newborns admitted to our unit at the University of Health Sciences, Zeynep Kamil Maternity and Children’s Training and Research Hospital, İstanbul, Turkey, during the study period. This retrospective study was approved by Zeynep Kamil Maternity and Children’s Training and Research Hospital Ethics Committee.

### 2.2. Data Collection

The database was extracted from electronic and paper patient files. We collected the following relevant neonatal and maternal data: gestational age, birth weight, gender, maternal age, maternal risk factors, mode of delivery, Apgar scores, hematological parameters and C-reactive protein (CRP) values at the sepsis diagnosis, maximal CRP (CRP max) value during EOS, causative pathogens and antibiotic susceptibility patterns.

### 2.3. Study Definitions

Early-onset sepsis was defined as positive blood culture with clinical and laboratory findings consistent with sepsis in the first 72 h of life [1]. The cases with white blood cell and neutrophil values outside the normal limits and/or CRP values above 1mg/dL were considered as suspicious for sepsis [10].

Blood samples for culture were drawn from a peripheral vein with antiseptic technique by experienced neonatal nurses. Povidone iodine (Isosol, Merkez ilac, İstanbul, Turkey) was used for skin preparation. After scrubbing the venopuncture site 3 times with povidone iodine and allowing the area to dry, blood samples were drawn. At least 1 mL of blood sample per blood culture bottle was inoculated. Blood cultures were analyzed with fully automated BacT/Alert 3D (BioMérieux, Durham, NC, USA) microbiological detection systems. Antibiotic susceptibility was determined by using the Kirby–Bauer disk diffusion test. Coagulase-negative *Staphylococcus*, *Corynebacterium* sp., *Diphtheroids* ssp., *Lactobacillus* sp., *Micrococcus* sp., *Stomatococcus* sp., and *Bacteroides* sp. grown only in a single blood culture and isolation of more than one microorganism in a blood culture specimen were considered as contaminants [1]. Pathogens were grouped into two categories: Gram-positive and Gram-negative. Sepsis foci, meningitis, urinary tract infection, pneumonia, and conjunctivitis were diagnosed if the EOS pathogen grew in cerebrospinal fluid, urine, tracheal aspirate, and conjunctiva cultures, respectively. Severe neutropenia was defined as a neutrophil count <500/μL.

Intraventricular hemorrhage (IVH) was diagnosed and graded by expert radiologists according to the modified Volpe classification [11]. Necrotizing enterocolitis (NEC) was staged by modifying Bell’s criteria [12]. Diagnosis of hemodynamically significant patent ductus arteriosus (PDA) was made according to echocardiographic measurements that show the size of PDA and magnitude of the ductal steal [13].

Maternal risk factors were obtained from mothers’ obstetrics medical records. Prolonged rupture of membranes (PROM) was defined as rupture of membranes more than 18 h before birth [1]. The diagnosis of clinical chorioamnionitis was made by a responsible obstetrician.

Early-onset sepsis-related mortality was defined as death within 7 days after bacterial growth in the blood culture. The empirical antibiotic therapy in our unit is the combination of ampicillin and gentamicin.

### 2.4. Statistical Analysis

SPSS version 20.0 (SPSS, Chicago, IL, USA) was used for statistical analysis, and Microsoft Office Excel was used to prepare tables and graphics. The Shapiro–Wilk test and histograms were used to test the normal distribution of data. Descriptive statistics were presented as percentages and mean ± standard deviation or median (interquartile range 25–75 percentiles) based on the normality of data distribution. Categorical data were analyzed using the chi-squared test. Mann–Whitney U test and Student’s *t*-test were used for comparisons between groups according to distribution. For the multivariate analysis, the possible factors identified with univariate analyses were further entered into the logistic regression analysis to determine predictors of patient outcome. Hosmer-Lemeshow goodness of fit statistics were used to assess the model fit. *p* < 0.05 was considered to be significant.

## 3. Results

### 3.1. Pathogen Distribution

During the study period, 56,278 deliveries took place in our hospital and 8229 of them were admitted to our NICU. Culture-proven EOS was detected in 101 (0.12%) patients among NICU admissions. The median gestational age and birth weight of patients was 36 weeks (IQR = 31–39) and 2700 g (IQR = 1542.5–3365), respectively. Of these, 54 (53.4%) patients were born as preterm (<37 weeks) and 14 (13.8%) were born as extremely preterm (<28 weeks) infants.

Out of 101 culture-proven EOS, 56 (55.4%) were Gram-positive and 45 (44.5%) were Gram-negative sepsis. The most common isolated microorganism was *E. coli* (28.7%, *n* = 29), followed by GBS (16.8%, *n* = 17) and *S. aureus* (15.8%, *n* = 16). All the etiologic agents of 101 EOS cases are shown in Table 1.

### 3.2. Clinical Characteristics

The frequency of multiple pregnancies, PROM, and chorioamnionitis were higher and the first- and fifth-minute Apgar scores were lower in the Gram-negative EOS group, and differences were statistically significant (*p* = 0.01, *p* = 0.02, *p* = 0.01, *p* = 0.01, *p* = 0.01, respectively). There were 33 mothers with high vaginal swab culture. There were two instances of *E. coli* growth among these vaginal cultures. Details about patient demographics, maternal risk factors, perinatal data, newborn comorbidities, and the distribution between Gram-negative and Gram-positive groups are depicted in Table 2.

At the time of sepsis onset, CRP was more than 1 mg/dL in 46 (45.5%) sepsis episodes. In 15 (14.8%) patients, CRP was low but elevated by more than 1 mg/dL in the following days. There was a mean of 24 h (12–52 h) between the first CRP and the second CRP values of the patients. CRP remained low in 40 (39.6%) patients. There was no difference between CRP levels of Gram-negative and Gram-positive EOS groups. The mean platelet values were lower in the Gram-negative EOS group, and differences were statistically significant (*p* = 0.004). Laboratory values during EOS and distribution to Gram-positive and -negative groups are shown in Table 3.

The onset of sepsis symptoms and obtaining the blood culture were in the first 24 h in 62.6% (*n* = 63) of patients. Lumbar puncture (LP) was performed in 50 (49.5%) patients, and meningitis was diagnosed in seven (6.9%) patients with EOS. Due to clinical instability, LP could not be performed in 82% (*n* = 14) of deceased patients. Culture-proven sepsis foci were identified in 13 patients, and two patients had more than one focus. Data are depicted in Table 4.

### 3.3. Mortality

Seventeen patients died because of EOS, and the mortality rate was 16.8%. In terms of the etiologic agent, the mortality rate was 23.5% in GBS and 10.7% in all Gram-positive EOS, whereas it was 20.6% in *E. coli* and 24.4% in all Gram-negative EOS. In the deceased newborn group, the most common isolated organisms were *E. coli* (*n* = 6, 35.2%), GBS (*n* = 4, 23.5%), *Klebsiella pneumoniae* (*n* = 2, 11.7%) and *Enterobacter Cloaca* (*n* = 2, 11.7%).

Among deceased newborns, 88.2% were preterm (*n* = 15), and the mortality rate in preterm newborns was 27.8%. However, the mortality rate among early-term and full-term newborns was 4.3% (*p* = 0.002). The timing of death was 0–3 days in 64.7% (*n* = 11) and 4–7 days in 35.3% (*n* = 6) of deceased newborns.

The mean gestational age and birth weight and the median Apgar 1 and Apgar 5 scores were lower in the deceased group than the discharged group, and differences were statistically significant (*p* < 0.001, *p* = 0.007, *p* < 0.001 and *p* < 0.001, respectively). In laboratory values at the time of sepsis diagnosis, median leucocyte, neutrophil, and the mean platelet count were lower in the deceased group, and differences were statistically significant (*p* = 0.043, *p* < 0.001 and *p* = 0.014, respectively: Table 5). Severe neutropenia was found to be an independent risk factor for mortality in EOS after an adjustment made by adding gestational age, Apgar 1, Gram stain of pathogens, leukocytes, and platelet count to the logistic regression model (*p* = 0.001, OR = 14.4, CI 95%: 2.8–74).

### 3.4. Antibiotic Susceptibility

The most commonly isolated pathogen in blood cultures, *E. coli*, was sensitive to ampicillin in 27.6% of cases and gentamicin in 93.1% of cases. The second most common pathogen, GBS, was sensitive to penicillin G in 93.3% of cases and ampicillin in 87.5% of cases. Only one GBS isolate was resistant to ampicillin, and another GBS isolate was resistant to penicillin G. An ampicillin and gentamicin combination had enough antimicrobial coverage in 92.6% (*n* = 88/95) of cases that either ampicillin or gentamicin susceptibility was tested. The evaluation of ampicillin and gentamicin resistance over 2-year periods during the study is shown in Table 6. Although it was not statistically significant, there was a decreasing trend in gentamicin resistance over the years. Additionally, 8 (27.6%) of 29 *E. coli* isolates were found to be extended-spectrum ß-lactamase (ESBL)-producing. All ESBL-producing *E. coli* were gentamicin-susceptible, except one which was amikacin-susceptible.

Two *E. coli*, two *S. aureus*, one *Enterobacter cloacae*, one *Streptococcus viridans* and one *Streptococcus pneumoniae* isolate were resistant to empirical ampicillin–gentamicin combination therapy. In five of these patients, empiric therapy was substituted by broad spectrum antibiotics, and none of them have died. Two patients with *E. coli* and *Enterobacter cloacae* growth died in first day of life, hence antibiotic treatment could not be changed. Commonly tested antibiotics and their susceptibility in Gram-negative and Gram-positive EOS are given in Table 7.

## 4. Discussion

In this single-center study of a 7-year experience with more than 8000 admissions, the rate of culture-proven early-onset sepsis was 0.12%. Our results have shown that although the majority of causative agents were Gram-positive (55.4%), the most common isolated organism was *E. coli* (28.7%), followed by GBS (16.8%), *S. aureus* (15.8%), *S. viridans* (10.9%), and *K. Pneumoniae* (7.9%). In our study, EOS mortality is 16.8% in all study populations, 4.3% in term newborns, and 27.8% in premature newborns. There was no difference between Gram-positive and -negative groups in terms of mortality. Severe neutropenia was found to be an independent risk factor for mortality in this study. It was demonstrated that there is a low level of antibiotic resistance to the current recommended empirical antibiotic regimen of ampicillin and gentamicin, and this empirical therapy continues to have enough coverage for EOS in our population.

Screening and prophylaxis for GBS in pregnancy has dramatically decreased GBS-related EOS [1]. Although a recent study has shown that *E. coli* (36.6%) is the most commonly isolated pathogen followed by GBS (30.2%) in the USA in extremely preterm infants, GBS is still the most common causative agent of EOS in most developed countries [8]. On the contrary, in developing countries, Gram-negative bacteria such as *E. coli, Klebsiella* spp., *Enterobacter,* and *Acinetobacter* are responsible for the majority of EOS [6]. In a single-center study in Egypt, Gram-negative pathogens were responsible for 86% of EOS, and *Klebsiella pneumoniae* was the most common pathogen [14]. In a recent review of Khalil et al., EOS in Middle Eastern countries was more likely to be due to Gram-negative pathogens, and *E. coli* (20%) was the most common organism, followed by *Klebsiella* spp. (18%), GBS (14%) and *S. aureus* (11%) [6]. Although the majority of causative agents were Gram-positive, *E. coli* was the most commonly isolated microorganism in our study. There is a limited number of studies on neonatal sepsis incidence, etiological factors and antibiotic susceptibility in our country. Most of the studies include both early- and late-onset sepsis results, and antibiotic susceptibility has not been assessed. In a study conducted by Ozkan et al. including preterm infants, the most common cause of early- and late-onset sepsis was coagulase-negative *Staphylococci* [15]. In a study by Taskın et al., the most commonly isolated pathogen in EOS was *S. aureus*, followed by *E. coli* and coagulase-negative *Staphylococcus* [16]. Unlike the previous studies from our country, *E. coli* was the leading pathogen, and the frequency of GBS was found to be higher in our study. Since GBS frequency is not as frequent as in developed countries, we do not think that routine GBS screening should be performed on pregnant women in our country. In our hospital, a risk-based GBS screening strategy is used, and ampicillin is the drug of choice in GBS prophylaxis.

According to the results of our study, the ampicillin and gentamicin combination had enough antimicrobial coverage in 92.6% of cases that either ampicillin or gentamicin susceptibility was tested. Comparable with our results, Khalil et al. have shown that overall susceptibility to ampicillin/gentamicin and third-generation cephalosporin were 40% and 37%, respectively, in middle-income countries, versus 93% and 91%, respectively, in high-income countries in the Middle East [6]. In developed countries, susceptibility to empirical antibiotic therapy for EOS is much higher. In a study by Vatne et al., 98% of microbial isolates were susceptible to the empirical antibiotic regimen of benzylpenicillin and gentamicin in Norway [4]. In a cohort study by Stoll, 7.8% of *E. coli* isolates were resistant to both ampicillin and gentamicin. In the same study, it was reported that two preterm infants infected with strains resistant to both ampicillin and gentamicin had died [8]. In a secondary analysis of the same study, 7.3% of isolates were found to be non-susceptible to both ampicillin and gentamicin [17]. In our study, two *E. coli*, two *S. aureus*, one *Enterobacter cloacae*, one *Streptococcus viridans* and one *Streptococcus pneumoniae* isolate were resistant to empirical ampicillin–gentamicin therapy. In five of these patients, empiric therapy was substituted by broad spectrum antibiotics and all of them survived. Two premature patients with *E. coli* and *Enterobacter cloacae* growth died on first day of life. They had PROM and insufficient antenatal care. The American Academy of Pediatrics (AAP) suggests that the empirical addition of broader-spectrum antibiotic therapy may be considered for preterm infants who are severely ill and at the highest risk for Gram-negative EOS (such as infants with prolonged PROM and who are exposed to prolonged courses of antepartum antibiotic therapy) until culture results are available [18,19]. Although it was demonstrated that the empirical antibiotic regimen of ampicillin and gentamicin continues to have enough coverage for EOS in our population, risk factors defined by AAP should be considered in terms of the necessity of initiating broad-spectrum antibiotics, especially in preterm infants, and the antibiotic susceptibilities of EOS pathogens should be monitored with ongoing surveillance.

Although many improvements in neonatal care and sepsis treatment have been achieved, EOS mortality is still high, especially in premature newborns [4,8]. In our study, comparable with previous studies, EOS mortality is 16.8% in the whole study population, 4.3% in term infants and 27.8% in premature newborns. There were no differences between Gram-positive and -negative groups in terms of mortality. Mortality rates in GBS and *E. coli* EOS were similar in Stoll et al. and our study: 7 vs. 9% for GBS and 33 vs. 33% for *E. coli*, respectively [8].

Neutrophils are critical for newborns to fight against pathogens due to their lack of adaptive immunity [20]. Low neutrophil counts reflect depleted granulocyte reserves in the bone marrow, and it is an indicator of poor prognosis in neonatal sepsis [20]. A recent study revealed that neutrophil counts are a strong predictor of EOS [21]. Severe neutropenia was found to be an independent risk factor for EOS mortality after adjusting other possible variables in this study.

Our results revealed that only 46 (45.5%) patients had a CRP level of more than 1 mg/dL, and there were no differences between maximum CRP levels between Gram-positive- and Gram-negative-induced EOS, similar to previous studies on inflammatory markers in EOS [4]. There was no difference between two groups in terms of the time of onset of symptoms, either. However, the presence of chorioamnionitis, PROM, and a low Apgar score were significantly more common in patients with Gram-negative growth. This should be considered an important warning for the clinical evaluation of infants at risk of EOS.

Our study has several limitations. Approximately 8000 deliveries occur annually in our hospital, but all newborns with hospitalization indications cannot be admitted to our unit and some of them are referred to other hospitals from our delivery room. That is why this study is not population-based and EOS incidence could not be achieved from our data. Although the retrospective nature of this study might be assessed as a limitation, we were able to reach detailed data of all infant and mother dyads, including susceptibility patterns of causative microorganisms, antibiotic use, and other laboratory findings over the entire study period. We think that one of the strengths of the study is recruiting only patients with blood culture growth, which is the gold standard in the diagnosis of sepsis. However, we only collected CSF culture results from patients who had blood culture-proven EOS. Therefore, we did not assess early-onset meningitis in newborns with sterile blood culture. One of the limitations of our study was a lower rate of LP performance. It is well known that LP cannot be performed if an infant’s clinical condition is not stable enough and if there is bleeding abnormality. In a CDC surveillance by Schrag et al., meningitis was diagnosed in 4% of EOS cases, but only half of the cases were diagnosed by using CSF culture, reflecting the practical difficulties in performing LP in this vulnerable group of patients [22]. A vast majority of our patients were under the treatment of mechanical ventilation, and nearly 35% of patients had thrombocytopenia. Therefore, our LP rate was lower than expected.

In conclusion, EOS is still a significant cause of morbidity and mortality among newborns, especially premature infants. Severe neutropenia is an independent risk factor for mortality in newborns with EOS. Although it was demonstrated that the empirical antibiotic regimen of ampicillin and gentamicin continues to have enough coverage for EOS in our population, care should be taken in terms of risk factors for the necessity of broad-spectrum antibiotics, especially in preterm infants. The antibiotic susceptibilities of EOS pathogens should be monitored with ongoing surveillance.

## Figures and Tables

**Table 1:** Etiologic agents detected in blood culture of patients with early-onset sepsis.

	*n* = 101
Gram-positive ^1^	56 (55.4)
*Streptococcus agalactiae* ^1^	17 (16.8)
*Staphylococcus aureus* ^1^	16 (15.8)
*Streptococcus viridans* ^1^	11 (10.9)
*Streptococcus* spp. ^1^	5 (4.9)
*Enterococcus faecalis* ^1^	4 (3.9)
*Streptococcus pneumoniae* ^1^	1 (1)
*Bacillus* spp. ^1^	1 (1)
*Listeria monocytogenes* ^1^	1 (1)
Gram-negative ^1^	45 (44.6)
*Escherichia coli* ^1^	29 (28.7)
*Klebsiella pneumoniae* ^1^	8 (7.9)
*Enterobacter cloacae* ^1^	3 (2.9)
*Serratia marcescens* ^1^	1 (1)
*Acinetobacter baumanii complex* ^1^	1 (1)
*Moraxella* spp. ^1^	1 (1)
*Pantoea agglomerans* ^1^	1 (1)
*Morganella morganii* ^1^	1 (1)

^1^: *n* (%).

**Table 2:** Neonatal and maternal demographics of the patients according to blood culture growth.

	Total *n* = 101	Gram-Positive *n* = 56	Gram-Negative *n* = 45	*p*
Gender, female ^1^	44 (43.6)	25 (44.6)	19 (42.2)	0.8
Mode of delivery, VD ^1^	45 (44.6)	22 (39.3)	23 (51.1)	0.23
Multiple pregnancy ^1^	15 (14.9)	4 (7.1)	11 (24.4)	0.01
Gestational age, weeks ^2^	36 (31–39)	36 (32.5–39)	36 (28–38.5)	0.15
≥37 ^1^	47 (46.5)	27 (48.2)	20 (44.4)	0.08
28–36 ^1^	40 (39.6)	25 (44.6)	15 (39.6)	
<28 ^1^	14 (13.9)	4 (7.1)	10 (22.2)	
Birth weight, grams ^2^	2700 (1542.5–3365)	2740 (1755–3452.5)	2515 (1170–3275)	0.3
Maternal risk factors				
Maternal age, years ^3^	27.8 ± 6	27 ± 6.3	29 ± 5.5	0.07
Premature rupture of membranes ^1^	23 (22.8)	8 (14.3)	15 (33.3)	0.02
Chorioamnionitis ^1^	9 (8.9)	1 (1.8)	8 (17.8)	0.01
Oligohydroamniosis ^1^	11 (10.9)	4 (7.1)	7 (15.6)	0.21
Gestational diabetes ^1^	9 (8.9)	4 (7.1)	5 (11.1)	0.5
Preeclampsia ^1^	11 (10.9)	8 (14.3)	3 (6.7)	0.33
Delivery room data				
Apgar 1 ^2^	7 (5–8)	8 (6–8)	7 (4–8)	0.01
Apgar 5 ^2^	8 (8–9)	9 (8–9)	8 (7–9)	0.01
Meconium-stained ^1^	9 (8.9)	3 (5.4)	6 (13.3)	0.18
Neonatal comorbidities				
RDS ^1^	33 (32.7)	14 (25)	19 (42.2)	0.08
TTN ^1^	26 (25.7)	19 (33.9)	7 (15.6)	0.03
Pulmonary hypertension ^1^	12 (11.9)	7 (12.5)	5 (11.1)	0.83
Pneumothorax ^1^	4 (4)	0 (0)	4 (8.9)	0.03
Clinical pneumonia ^1^	14 (13.9)	10 (17.9)	4 (8.9)	0.19
Invasive ventilation ^1^	41 (40.6)	18 (32.1)	23 (51.1)	0.05
Non-invasive ventilation ^1^	47 (46.5)	31 (55.4)	16 (35.6)	0.04
NEC ≥ stage 2 ^1^	4 (4)	1 (1.8)	3 (6.7)	0.32
NEC surgery ^1^	2 (2)	1 (1.8)	1 (2.2)	1
Hemodynamically significant PDA ^1^	26 (25.7)	12 (21.4)	14 (31.1)	0.26
IVH ≥ grade 2 ^1^	10 (9.9)	4 (7.1)	6 (13.3)	0.33

^1^: *n* (%), ^2^: median (interquartile range), ^3^: mean ± standard deviation, VD: vaginal delivery, RDS: respiratory distress syndrome, TTN: transient tachypnea of newborn, NEC: necrotizing enterocolitis, PDA: patent ductus arteriosus, IVH: intraventricular hemorrhage.

**Table 3:** Laboratory values of patients at the time of EOS.

	Overall *n* = 101	Gram-Positive *n* = 56	Gram-Negative *n* = 45	*p*
Leucocyte ×1000, (/μL) ^1^	9.2 (4.2–18.1)	9.8 (4.4–17.3)	8.7 (3.8–18.6)	0.96
Neutrophil ×1000, (/μL) ^1^	4.5 (1–9.8)	4.7 (1.2–9.8)	4.5 (0.7–11.7)	0.36
Neutropenia ^2^	25 (24.8)	11 (19.6)	14 (31.1)	0.184
Severe neutropenia ^2^	12 (11.9)	5 (8.9)	7 (15.6)	0.306
Platelet ×1000, (/μL) ^3^	187 ± 79	207 ± 74	162 ± 80	0.004
Thrombocytopenia ^2^	35 (34.7)	13 (23.2)	22 (48.9)	0.007
CRP, (mg/dL) ^1^	0.9 (0.3–3.1)	0.9 (0.3–2.1)	1.1 (0.3–3.8)	0.39
CRP max, (mg/dL) ^1^	2.2 (0.4–5.1)	1.4 (0.3–5.2)	2.4 (0.7–5.3)	0.61

^1^: median (interquartile range), ^2^: *n* (%), ^3^: mean ± standard deviation, CRP: c-reactive protein.

**Table 4:** Time of onset of symptoms and sepsis foci in patients.

	Total *n* = 101	Gram-Positive *n* = 56	Gram-Negative *n* = 45	*p*
Onset of symptoms				0.85
0–24 hours ^1^	62 (61.4)	35 (62.5)	27 (60)	
24–48 hours ^1^	19 (18.8)	11 (19.6)	8 (17.8)	
48–72 hours ^1^	20 (19.8)	10 (17.9)	10 (22.2)	
Sepsis foci ^1^	13 (12.9)	6 (10.7)	7 (15.6)	0.69
Meningitis ^1^	7 (7)	4 (7.2)	3 (6.6)	
Pneumonia ^1^	6 (6)	2 (3.6)	4 (8.9)	
Urinary tract ^1^	1 (1)	1 (1.8)	0 (0)	
Conjunctivitis ^1^	1 (1)	0 (0)	1 (2.2)	

^1^: *n* (%).

**Table 5:** Characteristics of survived and deceased newborns in early-onset sepsis.

	Survived *n* = 84	Deceased *n* = 17	*p*
Gender, female ^1^	37 (44)	7 (41.2)	0.828
Gestational age, weeks ^2^	37 (33–39)	31.5 (26.5–35.7)	0.001
≥37 ^1^	45 (53.6)	2 (11.8)	0.002
28–36 ^1^	31 (36.9)	9 (52.9)	
<28 ^1^	8 (9.5)	6 (35.3)	
Birth weight, grams ^2^	2915 (1885–3480)	1580 (1058–3120)	0.007
Mode of delivery, NVD ^1^	36 (42.9)	9 (52.9)	0.446
Maternal age, years ^2^	27.5 (23–32)	29 (21–32)	0.971
Premature rupture of membranes ^1^	21 (25)	2 (11.8)	0.235
Chorioamnionitis ^1^	6 (7.1)	3 (17.6)	0.166
Oligohydroamniosis ^1^	9 (10.7)	2 (11.8)	0.899
Gestational diabetes ^1^	6 (7.1)	3 (17.6)	0.166
Preeclampsia ^1^	9 (10.7)	2 (11.8)	0.899
Apgar 1 ^2^	8 (6–8)	4 (3–5)	<0.001
Apgar 5 ^2^	9 (8–9)	7 (6–8)	<0.001
Gram-negative ^1^	34 (40.5)	11 (64.7)	0.067
Leucocyte ×1000, (/μL) ^2^	10.9 (5.4–18.6)	3.93 (2–6.7)	0.043
Neutrophil ×1000, (/μL) ^2^	5.515 (1.28–12.3)	0.385 (0.16–1.45)	<0.001
Neutropenia ^1^	16 (19)	9 (53)	0.003
Severe neutropenia ^1^	4 (4.8)	8 (47.1)	<0.001
Platelet ×1000, (/μL) ^2^	204 (125–274.5)	148 (117–175.5)	0.014
Thrombocytopenia ^1^	25 (29.8)	10 (58.8)	0.022
CRP, (mg/dL) ^2^	0.8 (0.3–3.3)	1.1 (0.5–2.1)	0.374
CRP max, (mg/dL) ^2^	2.1 (0.3–5.7)	1.6 (1–4.3)	0.545

^1^: *n* (%), ^2^: median (interquartile range), NVD: normal vaginal delivery, CRP: C-reactive protein.

**Table 6:** The evaluation of antibiotic resistance over 2 year-periods during the study.

	Overall	2013–2014	2015–2016	2017–2018	2019–2020	*p*
Ampicillin or gentamicin resistance ^1^	7/95 (7.4)	4/27 (14.8)	0/21 (0)	2/22 (9.1)	1/25 (4)	0.224
Ampicillin resistance ^1^	40/73 (54.8)	16/25(64)	7/15 (46.7)	7/13 (53.8)	10/20 (50)	0.695
Gentamicin resistance ^1^	9/69 (13)	6/26 (23.1)	2/14 (14.3)	1/13 (7.7)	0/16 (0)	0.168
ESBL-producing *E. coli* ^1^	8/29 (27.6)	1/9 (11.1)	1/3 (33.3)	3/7 (42.9)	3/10 (30)	0.548

^1^: *n* (%), ESBL: extended-spectrum ß-lactamase.

**Table 7:** Commonly tested antibiotics for susceptibility.

Antibiotics Tested for Gram-Positive Organisms	Sensitive	Resistant	Tested for Susceptibility *n* = 56
Ampicillin ^1^	22 (71)	9 (29)	31 (55.4)
Gentamicin ^1^	21 (77.8)	6 (22.2)	27 (48.2)
Penicillin G ^1^	25 (55.6)	20 (44.4)	45 (80.4)
Ciprofloxacin ^1^	23 (85.2)	4 (14.8)	27 (48.2)
Cefotaxime ^1^	13 (81.2)	3 (18.8)	16 (28.6)
Ceftriaxone ^1^	24 (82.8)	5 (17.2)	29 (51.8)
Clindamycin ^1^	29 (67.4)	14 (32.6)	43 (76.8)
Erythromycin ^1^	24 (58.5)	17 (41.5)	41 (73.2)
Vancomycin ^1^	49 (98)	1 (2)	50 (89.3)
**Antibiotics Tested for Gram-Negative Organisms**	**Sensitive**	**Resistant**	**Tested for Susceptibility *n* = 45**
Ampicillin ^1^	11 (26.2)	31 (73.8)	42 (93.3)
Gentamicin ^1^	39 (92.9)	3 (7.1)	42 (93.3)
Amikacin ^1^	38 (100)	0 (0)	38 (84.4)
Meropenem ^1^	40 (97.6)	1 (2.4)	41 (91.1)
Ciprofloxacin ^1^	37 (90.2)	4 (9.8)	41 (91.1)
Cefuroxime ^1^	27 (65.9)	14 (34.1)	41 (91.1)
Ceftriaxone ^1^	31 (73.8)	11 (26.2)	42 (93.3)
Ceftazidime ^1^	23 (71.9)	9 (28.1)	32 (71.1)
Cefepime ^1^	29 (74.4)	10 (25.6)	39 (86.7)
Piperacillin-tazobactam ^1^	27 (87.1)	4 (12.9)	31 (68.9)

^1^: *n* (%).

## Data Availability

The datasets generated and analyzed for the study are available from the corresponding author upon a reasonable request.
